# Predictors of e-cigarette discontinuation among a US national cross-sectional sample of adults who currently smoke

**DOI:** 10.18332/tid/224362

**Published:** 2026-10-16

**Authors:** Yufei Wang, Scott R. Weaver, Jiapa Kutulike, Jidong Huang

**Affiliations:** 1 School of Public Health, Georgia State UniversityAtlantaUnited States

**Keywords:** e-cigarette discontinuation, cigarette, flavor, harm perception, predictors

## Abstract

**Introduction:**

A substantial proportion of people who smoke cigarettes have used e-cigarettes to reduce or quit smoking. However, there are many transitions to either using both e-cigarettes and cigarettes, or returning to smoking cigarettes exclusively. Limited research has focused on this population. This study aims to examine whether e-cigarette characteristics, use patterns, and perceptions of e-cigarettes predict e-cigarette discontinuation among people who smoke cigarettes.

**Methods:**

Data were drawn from the Adult Consumers of Tobacco Study, a nationally representative cross-sectional study conducted in 2023. A total of 2644 US adults completed an online questionnaire. We conducted a secondary analysis of 1507 participants who currently smoked cigarettes and had previously used e-cigarettes or were currently using e-cigarettes at the time of the survey.

**Results:**

Use of two flavors (adjusted odds ratio, AOR=0.53; 95% CI: 0.37–0.75) and more than two flavors (AOR=0.42; 95% CI: 0.29–0.62) was associated with lower odds of e-cigarette discontinuation. Compared with tobacco flavor use, use of fruit flavor (AOR=0.58; 95% CI: 0.42–0.80) and candy, dessert, chocolate, or other sweet flavors (AOR=0.57; 95% CI: 0.41–0.79) was also associated with lower odds of e-cigarette discontinuation. Additionally, greater perceived absolute harm of e-cigarettes (somewhat vs not at all/slight harmful, AOR=2.00; 95% CI: 1.20–3.35; very/extremely vs not at all/slight harmful, AOR=4.25; 95% CI: 2.60–6.99) and greater perceived relative harm of e-cigarettes compared with conventional cigarettes (about the same level of harm vs much less/less harmful, AOR=2.60; 95% CI: 1.86–3.66, more/much more harmful vs much less/less harmful, AOR=3.45; 95% CI: 2.22–5.46) were associated with higher odds of e-cigarette discontinuation.

**Conclusions:**

Flavors were associated with e-cigarette continuation, and perceptions of e-cigarette harm were associated with e-cigarette discontinuation among people who smoke cigarettes. Additional studies are needed to provide stronger evidence regarding these associations.

## Introduction

The evidence supporting the effectiveness of e-cigarettes (also known as electronic nicotine delivery devices or vapes) as a cigarette smoking cessation method is growing but remains limited^1-3^. However, e-cigarettes have often been marketed as a less harmful alternative to conventional cigarettes^4,5^. A substantial proportion of adults who smoke cigarettes have used e-cigarettes in an attempt to reduce or quit smoking^6,7^. However, many of these individuals continue smoking cigarettes and transition into either dual use of cigarettes and e-cigarettes or return to exclusive cigarette smoking^8^. Understanding factors associated with e-cigarette continuation or discontinuation among adults who smoke cigarettes may help explain why some individuals revert to exclusive cigarette use.

Several studies have examined various factors associated with e-cigarette discontinuation among people who have ever used e-cigarettes (regardless of cigarette use). One study using a nationally representative sample found that greater perceived harm of nicotine was associated with a higher likelihood of discontinuing e-cigarette use^9^. Another longitudinal study reported that greater perception of e-cigarette harm, shorter duration of e-cigarette use, and lower preferred nicotine concentration were predictors of e-cigarette discontinuation^10^.

However, research focusing specifically on individuals who smoke cigarettes and have tried e-cigarettes remains limited, and the predictors of e-cigarette discontinuation in this population may differ substantially from those among people who use e-cigarettes regardless of cigarette use. Prior studies suggested that discontinuation of e-cigarette use appears to occur more frequently among individuals whose use was not goal-oriented (e.g. curiosity) than among those who used e-cigarettes with a specific goal, such as quitting smoking^11,12^. Compared with the broader population of people who use e-cigarettes^13^, individuals who smoke cigarettes are more likely to report using e-cigarettes to reduce or quit smoking^6,14^ and therefore may be less likely to discontinue e-cigarette use. Consequently, predictors of e-cigarette discontinuation among people who smoke cigarettes may not be adequately captured by findings derived from studies of people who use e-cigarettes.

The aim of this study was to examine predictors of e-cigarette discontinuation among people who smoke cigarettes and have previously used e-cigarettes, focusing on e-cigarette characteristics, use patterns, and perceptions of e-cigarettes. The findings may provide additional evidence on factors associated with e-cigarette discontinuation among this understudied population.

## Methods

### Study design and sample

The data used in this study were derived from the Adult Consumers of Tobacco Study, a nationally representative cross-sectional study of adults who currently smoke cigarettes in the US, conducted by Georgia State University (GSU). Between August and October 2023, the National Opinion Research Center (NORC) at the University of Chicago, on behalf of GSU, recruited 2644 US adults aged ≥18 years who currently smoke cigarettes from its AmeriSpeak probability-based panel and a non-probability panel. Survey weights were calibrated to the AmeriSpeak probability panel to improve representativeness. Data were collected through an online questionnaire to obtain information on tobacco use behaviors and perceptions of various nicotine products. Specifically, if invited, participants can take the survey online through the password-protected AmeriSpeak Mobile App, the password-protected AmeriSpeak Web portal, or by following a link in the e-mail invitation sent to them. Participants were offered the cash equivalent of $4 for completing the survey. The final sample included set quotas of current e-cigarette users, former e-cigarette users, and those who have never used e-cigarettes.

In the current study, we conducted a secondary analysis focusing on 1594 adults who currently smoke cigarettes and have used e-cigarettes previously. After excluding participants with missing values in the outcome, independent, and covariate variables, a total of 1507 participants were included in the final analysis.

### Outcome variable

E-cigarette use status was determined using two survey questions: ‘Have you ever used a nicotine e-cigarette/vape, even one or two times?’ and ‘Do you now use a nicotine e-cigarette/vape every day, some days, or not at all?’. Participants who answered ‘Yes’ to the first question and ‘Every day’ or ‘Some days’ to the second question were categorized into the current use group, while those who answered ‘Yes’ to the first question and ‘Not at all’ to the second question were categorized into the discontinuation group.

### Predictor variables

Based on prior literature, e-cigarette characteristics and use patterns, and perceptions of e-cigarettes were considered likely to influence the discontinuation of e-cigarette use^10,15^. In the current study, e-cigarette characteristics and use patterns included: 1) the number of e-cigarette flavors used or currently being used, categorized into three groups: 1 flavor, 2 flavors, ≥3 flavors; 2) use (ever or current) of specific flavor categories was assessed as separate dichotomous variables (yes, no), including mint, wintergreen, or menthol; fruit; candy, dessert, chocolate, or other sweets; clove or spice; alcoholic drink flavors (e.g. wine, cognac, margarita, or other cocktails); and tobacco flavor; 3) nicotine concentration (mg/mL), categorized into five groups: 1–6 or 0.1–0.6%, 7–17 or 0.7–1.7%, 18–39 or 1.8–3.9%, ≥40 or ≥4.0%, and ‘don’t know’; 4) use (ever or current) of a rechargeable device, defined as a reusable e-cigarette device powered by a rechargeable battery, categorized as yes or no; 5) use (ever or current) of a refillable device, defined as an e-cigarette device that can be refilled with e-liquid after use, categorized as yes or no; 6) lifetime e-cigarette use frequency, categorized as 2–10, 11–99, or ≥100 times; and 7) ever e-cigarette regular use. Participants were classified as regular users if they currently used e-cigarettes daily or if they responded ‘Yes’ to the question, ‘Have you ever used a nicotine e-cigarette/vape fairly regularly (at least several times a week or more)?’. All other participants were classified as non-regular users.

Perceptions of e-cigarettes included perceived absolute harm of e-cigarettes and perceived relative harm of e-cigarettes compared with conventional cigarettes. Perceived absolute harm of e-cigarettes was categorized into four levels: not at all/slightly harmful, somewhat harmful, very/extremely harmful, and don’t know. Perceived relative harm of e-cigarettes versus conventional cigarettes was also categorized into four levels: much less/less harmful, about the same level of harm, more/much more harmful, and don’t know.

### Covariates

Demographic variables include gender (male or female), age (18–29, 30–44, 45–59, or ≥60 years), race/ethnicity (White, non-Hispanic; Black, non-Hispanic; Hispanic; or other), household income ($) (<30000; 30000 to <60000; 60000 to <100000; ≥100000), marital status (married, never married, or widowed/divorced/separated), education level (high school graduate or lower; some college/associates degree or higher), region (Northeast, Midwest, South, or West), and metropolitan statistical area status (yes or no). Potential confounding factors include drinking alcohol within the past 30 days (yes or no), self-reported physical health (excellent, very good, good, fair, or poor), self-reported mental health, and cigarette use. Self-reported mental health was assessed using the six-item Kessler Psychological Distress Scale^16^. Scores range from 0 to 24, with higher scores indicating a greater likelihood of serious mental illness. All participants currently used cigarettes and were categorized into two groups based on their usage pattern: every day and some days. These demographic variables and potential confounding factors were included as covariates because they were theoretically considered likely to be associated with both the predictors (ie, e-cigarette characteristics and use patterns, and perceptions of e-cigarettes) and e-cigarette discontinuation^9,10^.

### Statistical analysis

Participants’ characteristics, e-cigarette characteristics and use patterns, and perceptions of e-cigarettes were analyzed by e-cigarette use status. Raw frequencies (weighted percentages) and weighted means (weighted standard deviations) were calculated. In addition, univariable weighted logistic regression models were used to examine the bivariate associations between participants’ characteristics and predictor variables and e-cigarette use status. Multivariable weighted logistic regression models were then constructed to estimate adjusted odds ratios (AORs) of e-cigarette characteristics, use patterns, and perceptions in relation to e-cigarette use status, adjusting for all covariates. In regression analyses of e-cigarette flavor use, the following dichotomous flavor variables were included simultaneously in the same model: mint, wintergreen, or menthol; fruit; candy, dessert, chocolate, or other sweets; clove or spice; alcoholic drink flavors. Tobacco flavor was used as the reference category for comparisons with other flavor types. All statistical tests were two-sided, with p<0.05 considered statistically significant. All analyses were conducted in December 2025 using R version 4.3.1.

## Results

### Participant characteristics and e-cigarette use status

Table 1 presents the relationships between sociodemographic, self-reported health, and cigarette use characteristics, with e-cigarette use status. Among 1507 participants, 68.6% used and subsequently discontinued e-cigarettes, and 44.7% were female. Participants aged 30–44 years (37.0%) constituted the largest age group, followed by those aged 45–59 years (28.6%), 18–29 years (17.8%), and ≥60 years (16.6%). In addition, female participants (crude odds ratio, OR=1.37; 95% CI: 1.05–1.79), older individuals (30–44 years vs 18–29 years: OR=2.18; 95% CI: 1.53–3.13; 45–59 years vs 18–29 years: OR=3.72; 95% CI: 2.51–5.55; ≥60 years vs 18–29 years: OR=5.24; 95% CI: 3.25–8.65), those with better mental health (OR=0.96; 95% CI: 0.94–0.98), and individuals smoking cigarette every day (vs some days, OR=2.32; 95% CI: 1.75–3.08) had higher odds of discontinuing e-cigarette use. By contrast, Hispanic participants (vs non-Hispanic White, OR=0.59; 95% CI: 0.38–0.90) and individuals who were never married (vs married, OR=0.48; 95% CI: 0.35–0.65) had lower odds of discontinuing e-cigarette use.

**Table 1:** Participant characteristics stratified by e-cigarette use status among US adults who currently smoke cigarettes, Adult Consumers of Tobacco Study (ACTS), 2023 (N=1507)

Characteristics	Overall (N=1507)	Current use (N=803)	Discontinuation (N=704)	OR (95% CI)
	n (wgt. %)			
**Gender**				
Male (ref.)	638 (55.3)	350 (60.6)	288 (52.9)	1
Female	869 (44.7)	453 (39.4)	416 (47.1)	**1.37 (1.05–1.79)**
**Age** (years)				
18–29 (ref.)	216 (17.8)	163 (29.9)	53 (12.3)	1
30–44	611 (37.0)	368 (39.8)	243 (35.7)	**2.18 (1.53–3.13)**
45–59	358 (28.6)	156 (21.0)	202 (32.1)	**3.72 (2.51–5.55)**
≥60	322 (16.6)	116 (9.3)	206 (20.0)	**5.24 (3.25–8.65)**
**Race/ethnicity**				
White, non-Hispanic (ref.)	1048 (75.3)	544 (71.9)	504 (76.8)	1
Black, non-Hispanic	186 (9.5)	102 (8.0)	84 (10.2)	1.20 (0.76–1.96)
Hispanic	185 (9.7)	103 (13.0)	82 (8.1)	**0.59 (0.38–0.90)**
Other	88 (5.5)	54 (7.2)	34 (4.8)	0.63 (0.36–1.09)
**Household income** ($)				
<30000 (ref.)	566 (40.6)	289 (38.0)	277(41.9)	1
30000 to <60000	488 (30.4)	265 (31.9)	223 (29.7)	0.85 (0.62–1.16)
60000 to <100000	281 (17.8)	152 (17.8)	129 (17.8)	0.90 (0.62–1.32)
≥100000	172 (11.2)	97 (12.3)	75 (10.7)	0.79 (0.51–1.23)
**Marital status**				
Married (ref.)	486 (30.9)	242 (23.9)	244 (34.1)	1
Never married	608 (44.0)	373 (56.3)	235 (38.4)	**0.48 (0.35–0.65)**
Widowed/divorced/ separated	413 (25.1)	188 (19.9)	225 (27.5)	0.97 (0.66–1.42)
**Education level**				
High school graduate or lower (ref.)	505 (49.9)	255 (52.2)	250 (48.9)	1
Some college/ associates degree or higher	1002 (50.1)	548 (47.8)	454 (51.1)	1.14 (0.88–1.49)
**Region**				
Northeast (ref.)	196 (13.6)	98 (10.3)	98 (15.1)	1
Midwest	415 (25.2)	221 (25.7)	194 (24.9)	0.66 (0.41–1.05)
South	628 (42.0)	337 (43.5)	291 (41.3)	0.65 (0.42–1.00)
West	268 (19.3)	147 (20.5)	121 (18.7)	0.62 (0.38–1.01)
Metropolitan statistical area status^*^	1226 (81.0)	658 (81.8)	568 (80.6)	0.93 (0.66–1.29)
Drinking alcohol within the past 30 days^*^	906 (59.6)	490 (59.1)	416 (59.8)	1.03 (0.79–1.35)
**Self-reported physical health**				
Excellent (ref.)	70 (3.8)	46 (5.3)	24 (3.1)	1
Very good	263 (17.4)	155 (20.0)	108 (16.2)	1.36 (0.67–2.73)
Good	688 (44.0)	365 (42.7)	323 (44.6)	1.74 (0.89–3.37)
Fair	390 (27.9)	194 (26.3)	196 (28.6)	1.81 (0.91–3.57)
Poor	96 (6.9)	43 (5.8)	53 (7.4)	2.15 (0.94–4.92)
**Self-reported mental health**, wgt. mean (wgt. SD)	7.49 (5.99)	8.46 (6.04)	7.05 (5.91)	**0.96 (0.94–0.98)**
**Cigarette use**				
Some days (ref.)	402 (28.2)	268 (40.4)	134 (22.6)	1
Every day	1105 (71.8)	535 (59.6)	570 (77.4)	**2.32 (1.75–3.08)**

* For binary variables coded as Yes/No, only the ‘Yes’ category is presented; ‘No’ was the reference group. For other categorical variables, all categories were shown. wgt: weighted. SD: standard deviation. OR: crude odds ratio. CI: confidence interval. ref: reference.

### E-cigarette characteristics, use patterns, and use status

Table 2 shows the associations between e-cigarette characteristics and use patterns with e-cigarette use status. Using two (vs one flavor, OR=0.40, 95% CI: 0.29–0.55) or more than two e-cigarette flavors (vs one flavor, OR=0.29; 95% CI: 0.20–0.40) was associated with lower odds of discontinuation of e-cigarette use. Compared with tobacco flavor, using fruit flavor (OR=0.49; 95% CI: 0.37–0.67), candy, dessert, chocolate, or other sweet flavors (OR=0.49; 95% CI: 0.36–0.67), and clove or spice flavors (OR=0.49; 95% CI: 0.24–0.99) was associated with lower odds of discontinuation of e-cigarette use. Individuals who used rechargeable devices (OR=0.64; 95% CI: 0.45–0.91), those who ever used e-cigarette regularly (OR=0.15; 95% CI: 0.11–0.21), and those with higher lifetime frequency of e-cigarette use (11–99 times vs 2–10 times, OR=0.30; 95% CI: 0.19–0.46; ≥100 times vs 2–10 times, OR=0.07; 95% CI: 0.05–0.11) were less likely to discontinue e-cigarette use.

**Table 2:** E-cigarette characteristics and use patterns stratified by e-cigarette use status among US adults who currently smoke cigarettes, Adult Consumers of Tobacco Study (ACTS), 2023 (N=1507)

Characteristics	Current use (N=803)	Discontinuation (N=704)	OR (95% CI)
	n (wgt. %)		
**Number of e-cigarette flavor categories used**			
1 (ref.)	347 (38.8)	463 (64.8)	1
2	251 (31.2)	147 (20.9)	**0.40 (0.29–0.55)**
≥3	205 (30.0)	94 (14.3)	**0.29 (0.20–0.40)**
Use of specific flavor categories^*^			
Tobacco flavor (ref.)	220 (25.9)	296 (44.2)	1
Mint/wintergreen/menthol flavors	157 (21.4)	93 (13.8)	0.76 (0.53–1.10)
Fruit flavor	479 (64.4)	265 (39.0)	**0.49 (0.37–0.67)**
Candy/dessert/chocolate/other sweet flavors	337 (48.7)	160 (24.1)	**0.49 (0.36–0.67)**
Clove/spice flavors	34 (6.7)	21 (2.5)	**0.49 (0.24–0.99)**
Alcoholic drink flavor	52 (7.9)	16 (2.3)	0.57 (0.28–1.11)
**Nicotine concentration** (mg/mL)			
1–6 or 0.1–0.6% (ref.)	160 (22.2)	58 (8.7)	1
7–17 or 0.7–1.7%	120 (13.2)	65 (9.4)	**1.82 (1.09–3.05)**
18–39 or 1.8–3.9%	104 (13.2)	44 (6.1)	1.18 (0.68–2.03)
≥40 or ≥4.0%	119 (18.7)	32 (5.4)	0.74 (0.44–1.26)
Don’t know	300 (32.7)	505 (70.4)	**5.50 (3.69–8.22)**
**Use of a rechargeable device**			
No (ref.)	146 (15.8)	154 (21.1)	1
Yes	647 (82.8)	492 (71.0)	**0.64 (0.45–0.91)**
Don't know	10 (1.4)	58 (7.9)	**4.33 (1.73–13.83)**
**Use of a refillable device**			
No (ref.)	371 (43.1)	281 (41.5)	1
Yes	408 (52.5)	350 (48.4)	0.96 (0.73–1.26)
Don't know	24 (4.3)	73 (10.1)	**2.43 (1.37–4.64)**
Ever e-cigarette regular use^*^	632 (82.5)	298 (42.1)	**0.15 (0.11–0.21)**
**Lifetime e-cigarette use** (times)			
2–10 (ref.)	96 (9.7)	314 (40.9)	1
11–99	296 (32.7)	288 (41.6)	**0.30 (0.19–0.46)**
≥100	411 (57.6)	102 (17.5)	**0.07 (0.05–0.11)**

* ‘Yes’ category is presented; ‘No’ was the reference group. For other categorical variables, all categories are shown. wgt.: weighted. OR: crude odds ratio. CI: confidence interval. ref: reference.

### Perceptions of e-cigarette and e-cigarette use status

Table 3 presents the associations between perceptions of e-cigarettes and e-cigarette use status. Compared with those who perceived e-cigarettes as not at all or slightly harmful, individuals who perceived them as somewhat harmful (OR=1.91; 95% CI: 1.19–3.08) or very or extremely harmful (OR=4.20; 95% CI: 2.67–6.68) had higher odds of discontinuing e-cigarette use. Similarly, compared with those who perceived e-cigarettes as less harmful than conventional cigarettes, individuals who perceived e-cigarettes as about the same harmful (OR=2.44; 95% CI: 1.79–3.32) or more or much more harmful than cigarettes (OR=3.00; 95% CI: 2.00–4.59) were more likely to discontinue e-cigarette use.

**Table 3:** Perceptions of e-cigarette stratified by e-cigarette use status among US adults who currently smoke cigarettes, Adult Consumers of Tobacco Study (ACTS), 2023 (N=1507)

	Current use (N=803)	Discontinuation (N=704)	OR (95% CI)
	n (wgt. %)		
**Perception of e-cigarette harm**			
Not at all/slightly harmful (ref.)	131 (15.8)	41 (5.6)	1
Somewhat harmful	266 (35.6)	158 (24.1)	**1.91 (1.19–3.08**)
Very/extremely harmful	351 (40.4)	438 (60.3)	**4.20 (2.67–6.68**)
Don’t know	55 (8.2)	67 (10.0)	**3.41 (1.87–6.34**)
**Perception of e-cigarette vs cigarette harm**			
Much less or less harmful (ref.)	368 (45.1)	155 (22.9)	1
About the same level of harm	270 (35.8)	320 (44.3)	**2.44 (1.79–3.32**)
More or much more harmful	104 (12.5)	130 (19.1)	**3.00 (2.00–4.59**)
Don’t know	61 (6.6)	99 (13.7)	**4.10 (2.48–7.03**)

wgt: weighted. OR: crude odds ratio. CI: confidence interval. ref: reference.

### Multivariable regression models

Figure 1 displays the odds ratios of e-cigarette discontinuation in models controlling for covariates. Compared with individuals using one flavor, those who used two (adjusted odds ratio, AOR=0.53; 95% CI: 0.37–0.75) and more than two flavors (AOR=0.42; 95% CI: 0.29–0.62) were less likely to discontinue using e-cigarettes. Compared with tobacco flavor use, use of fruit flavor (AOR=0.58; 95% CI: 0.42–0.80), and candy, dessert, chocolate, or other sweet flavors (AOR=0.57; 95% CI: 0.41–0.79) had lower odds of discontinuing e-cigarette use. Individuals who ever used e-cigarettes regularly (AOR=0.17; 95% CI: 0.12–0.24) and those with greater lifetime e-cigarette use (11–99 times vs 2–10 times, AOR=0.29, 95% CI: 0.19–0.45; ≥100 times vs 2–10 times, AOR=0.08; 95% CI: 0.05–0.12) were less likely to discontinue using e-cigarette. Nicotine concentration levels were not significantly associated with e-cigarette discontinuation, whereas individuals who reported ‘don’t know’ (vs 1–6 mg/mL or 0.1–0.6%, AOR=5.13; 95% CI: 3.35–7.88) were more likely to discontinue e-cigarette use.

**Figure 1:**
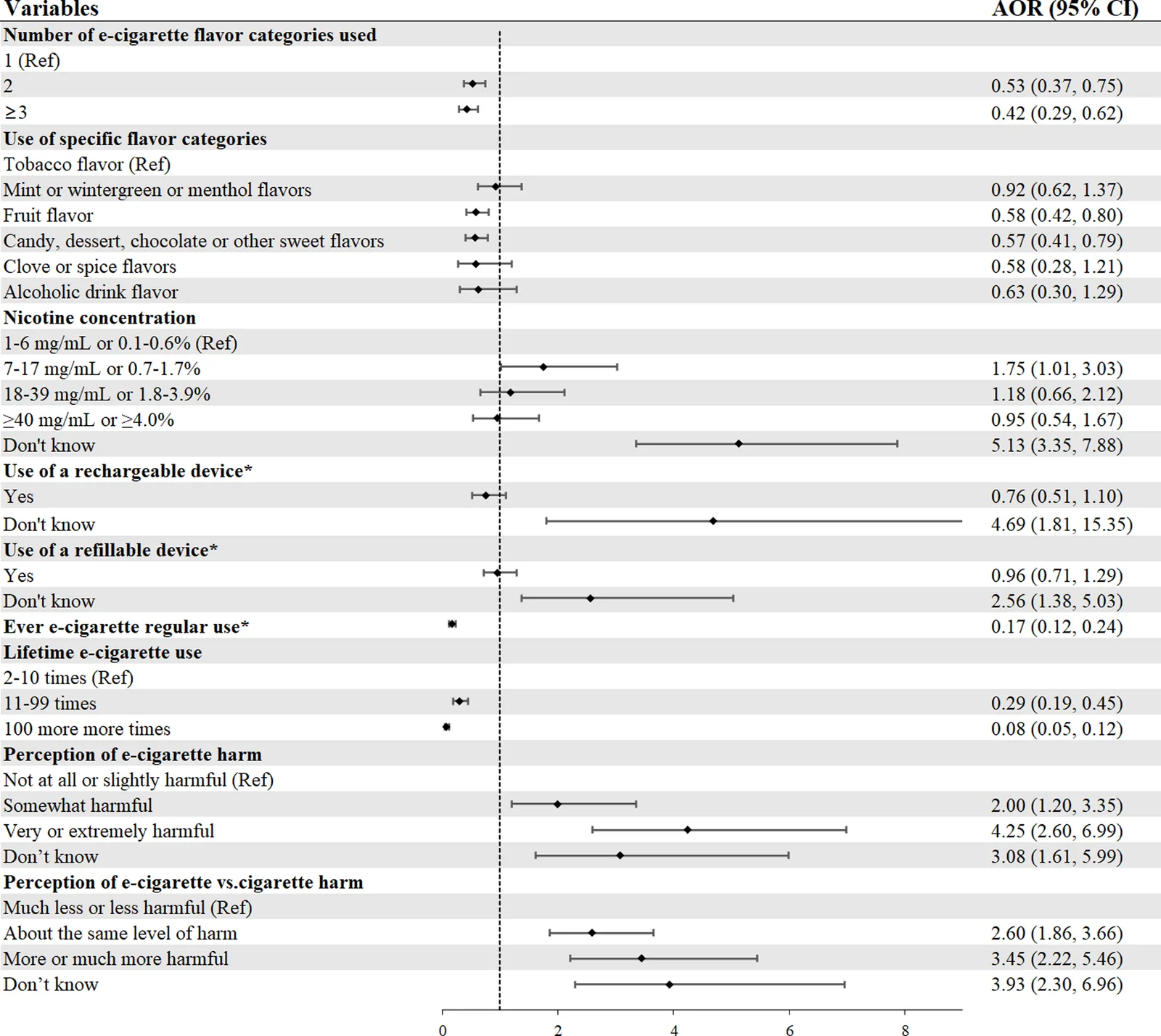
Predictors of e-cigarette discontinuation among US adults who currently smoke cigarette, Adult Consumers of Tobacco Study (ACTS), 2023 (N=1507)

AOR: adjusted odds ratio. CI: confidence interval. ref: reference. The e-cigarette use status variable was coded as: 1 = discontinuation and 0 = current use. Each model was adjusted for gender, age, race/ethnicity, household income, marital status, education level, region, metropolitan statistical area status, drinking alcohol within the past 30 days, self-reported physical health, self-reported mental health and cigarette use. *‘No’ was used as the reference group.

Regarding perceptions of e-cigarette harm, compared with individuals who perceived e-cigarettes as not at all or slightly harmful, those who perceived e-cigarettes as somewhat harmful (AOR=2.00; 95% CI: 1.20–3.35), very or extremely harmful (AOR=4.25; 95% CI: 2.60–6.99), and reported ‘don’t know’ (AOR=3.08; 95% CI: 1.61–5.99) had higher odds of discontinuing e-cigarette use. Similarly, compared with individuals who perceived e-cigarettes as not at all or slightly harmful than conventional cigarette, those who perceived e-cigarettes at about the same level of harm as conventional cigarettes (AOR=2.60; 95% CI: 1.86–3.66), more or much more harmful than conventional cigarettes (AOR=3.45; 95% CI: 2.22–5.46), and those who reported ‘don’t know’ (AOR=3.93; 95% CI: 2.30–6.96) were more likely to discontinue e-cigarette use.

## Discussion

To our knowledge, this study is among the first to examine e-cigarette characteristics, use patterns, and perceptions in relation to e-cigarette discontinuation among adults who currently smoke cigarettes. Our findings suggest that perceptions of the absolute and relative harm of e-cigarettes and e-cigarette flavor are significantly associated with e-cigarette discontinuation, whereas nicotine level is not significantly associated with e-cigarette discontinuation.

We found that individuals who perceived greater absolute harm and relative harm of e-cigarettes were more likely to discontinue e-cigarette use among people who currently smoke. In addition, individuals who selected ‘don’t know’ for the absolute or relative harm of e-cigarettes also tend to discontinue e-cigarette use. The first finding is in line with previous studies examining reasons for discontinuing e-cigarette use among people who smoke^17,18^, as well as studies investigating the relationships between perceived harms of e-cigarettes and e-cigarette discontinuation^10,19,20^. However, Kalan and Brewer^9^ found that neither absolute nor relative harm perceptions of e-cigarettes were significantly associated with e-cigarette discontinuation. The inconsistent findings may potentially be explained by differences in the study populations and the moderating effect of age^20,21^. The proportion of participants selecting ‘don’t know’ in the current study was larger than that reported in a similar nationally representative study^22^, which allowed us to estimate the relationship between this category and e-cigarette discontinuation. The positive associations between selecting ‘don’t know’ for absolute and relative perceived harms of e-cigarettes and e-cigarette discontinuation may be explained by experimental e-cigarette use. Specifically, some individuals may have tried e-cigarettes experimentally and discontinued use before forming clear perceptions about the harm of e-cigarettes. Greater attention to perceived e-cigarette harm among individuals who use both cigarettes and e-cigarettes may be needed in future studies.

The present results suggest that using multiple e-cigarette flavors and using fruit flavor or candy, dessert, chocolate, or other sweet flavors were associated with lower odds of e-cigarette discontinuation. There is limited research examining the direct relationship between e-cigarette flavors and e-cigarette discontinuation. However, the findings of the current study may be indirectly supported by research on e-cigarette flavors and continued e-cigarette use. Specifically, previous literature has indicated that flavor variety and non-tobacco flavors were associated with higher odds of continuing e-cigarette use^23-26^. One possible mechanism underlying the association between non-tobacco flavors and continued e-cigarette use is that fruit or sweet flavors provide a more satisfying e-cigarette use experience than tobacco flavors, making continued e-cigarette use more likely^27^. Longitudinal research on the effects of flavors on e-cigarette discontinuation is needed to better understand the role of flavor preferences in e-cigarette discontinuation behaviors over time.

The finding that nicotine level was not significantly associated with e-cigarette discontinuation, whereas individuals who reported not knowing their nicotine level were more likely to discontinue e-cigarette use, was inconsistent with several longitudinal studies suggesting that lower nicotine concentration predicts e-cigarette discontinuation^10,15^. Two reasons may explain this discrepancy. First, differences in study populations may play a role. The Pokhrel et al.^10^ study included individuals who used e-cigarettes at baseline regardless of cigarette use, and the participants in the Sobieski et al.^15^ study used e-cigarettes exclusively, whereas our study focused on people who currently smoked cigarettes and had used e-cigarettes. So, participants in our study may have been less reliant on e-cigarettes to achieve preferred nicotine levels due to a higher probability of concurrent cigarette use^28^. Second, participants in the discontinuation group may have had difficulty recalling the nicotine concentration they previously used, which could have attenuated true differences. This assumption was supported by the higher likelihood of ‘don’t know’ responses regarding nicotine concentration among participants who had discontinued e-cigarette use compared with those who were currently using e-cigarettes.

### Limitations

Several limitations of this study should be noted. First, due to the cross-sectional design, the observed associations cannot be interpreted as causal. Second, the data were self-reported, and recall bias is possible, particularly for e-cigarette–related information reported by individuals who were not using e-cigarettes at the time of the survey, potentially resulting in misclassification. Third, the majority of participants reported ‘don’t know’ regarding nicotine level, which may have obscured the true relationship between nicotine level and e-cigarette discontinuation. Future longitudinal research is needed to provide stronger evidence.

## Conclusions

E-cigarette flavors and perceptions of e-cigarette harm are associated with e-cigarette discontinuation among people who smoke cigarettes. Additional studies are needed to provide stronger evidence regarding these associations.
